# Polymicrobial Interactions Operative during Pathogen Transmission

**DOI:** 10.1128/mBio.01027-21

**Published:** 2021-05-18

**Authors:** Hannah M. Rowe, Jason W. Rosch

**Affiliations:** a Department of Microbiology, Oregon State University, Corvallis, Oregon, USA; b Department of Infectious Diseases, St. Jude Children’s Research Hospital, Memphis, Tennessee, USA; University of Texas Health Science Center at Houston

**Keywords:** coinfection, host-pathogen interactions, pathogenesis, transmission

## Abstract

Pathogen transmission is a key point not only for infection control and public health interventions but also for understanding the selective pressures in pathogen evolution. The “success” of a pathogen lies not in its ability to cause signs and symptoms of illness but in its ability to be shed from the initial hosts, survive between hosts, and then establish infection in a new host. Recent insights have shown the importance of the interaction between the pathogen and both the commensal microbiome and coinfecting pathogens on shedding, environmental survival, and acquisition of infection. Pathogens have evolved in the context of cooperation and competition with other microbes, and the roles of these cooperations and competitions in transmission can inform novel preventative and therapeutic strategies.

## INTRODUCTION

Transmission of pathogens is a multifactorial process by which a pathogen must be shed from an infected host, survive its transit between hosts, and then establish an infection in a new host. Understanding transmission dynamics of pathogens is key to control of endemic and epidemic infections. In addition, transmission is a point at which pathogens are under selective pressures, since the ultimate “success” of a pathogen is related not only to its ability to cause disease in its hosts but also to its ability to establish productive infections in new hosts. Pathogen transmission can be direct or indirect, it can involve many host species for a pathogen to undergo a complex life cycle, or it can be confined to a single host species. Transmission can occur over short distances of space or time, or a pathogen can spend a long time or distance in the air or associated with a biotic or abiotic surface between hosts.

Transmission factors can be pathogen-associated or host-associated. Some of the best understood pathogen transmission factors are bacterial toxins involved in inducing pathogen shedding, including the AB_5_ cholera (1) and pertussis (2) toxins, the cholesterol-dependent pore-forming toxin pneumolysin (3), and the viral enterotoxin nsP4 of rotavirus (4). Host-associated transmission factors can be immune or behavioral. Immune factors can alter both shedding and susceptibility. Naturally acquired or vaccine-induced immunity is mainly thought of as a way to prevent acquisition but can also alter shedding dynamics in the vaccinated host (5). Behavioral factors for humans are typically recreational and occupational exposures but can also be from direct pathogen control of behaviors, such as rabies’ induction of aggression and salivation and Toxoplasma gondii’s reduction of rodents’ fear responses (6, 7).

Using a more expansive definition of host, we can include the microbial members of the microbiome, mycobiome, and virome of the host and the roles that these play in shedding, environmental survival, and acquisition of pathogens. In addition, we explore recent insights into the roles of coinfecting pathogens on transmission. Beneficial and antagonistic interactions between microbes can occur in the infected host to alter shedding in the environment, in intermediate hosts to alter pathogen survival between hosts, and in the new host, changing susceptibility to acquisition.

## POLYMICROBIAL IMPACTS ON SHEDDING

The microbial community is typically considered to consist of the benign commensal occupants of the surfaces and mucosal sites of humans, plants, and animals, but it can also encompass pathogenic species. These pathogens could be asymptomatically colonizing or causing symptoms while infecting an organism. In addition to more intense signs and symptoms from coinfection, which can enhance shedding, infection by multiple infectious agents can increase the pathogen load at the mucosal site and therefore increase the likelihood and magnitude of shedding. Inflammatory molecules and signs and symptoms, such as coughing, sneezing, mucosal discharge, and diarrhea, induced by one pathogen can cause increased colonization density and shedding of other pathogens infecting the same mucosal site.

Infection with human influenza viruses (8, 9), respiratory syncytial virus (RSV) (10–13), or human rhinovirus (12) can increase the nasopharyngeal load of bacterial pathogens, including Streptococcus pneumoniae, Staphylococcus aureus, Moraxella catarrhalis, and Haemophilus influenzae. This effect is also seen in murine models (14, 15) and in humans (16–18) receiving live, attenuated influenza vaccine, with higher density and duration of colonization with pathogenic bacteria. Upper respiratory viral illnesses have been implicated in shedding of S. aureus in neonates (19) and in adults (20–22). The ability of influenza virus infection to facilitate increased transmission of S. pneumoniae has been seen in murine (23, 24) and ferret (25–27) models. Influenza infection can enhance inflammation and nasal secretions (28), impacting pneumococcal colonization density, shedding, and transmission (29, 30). Recent insights have shown that the coinfection of pneumococcus and influenza virus triggers shared interferon responses, enhancing shedding of both pathogens (31). Influenza A virus (IAV) infection can also trigger S. pneumoniae’s biofilm to planktonic transition (32, 33), which increases pathogenesis and could be a mechanism for enhanced shedding from the host.

The impact of bacterial colonization density on IAV transmission is mixed. Ferret models of depletion of respiratory flora or restoration of respiratory flora with S. pneumoniae did not alter viral load in the respiratory secretions of coinfected animals (34). However, in other studies, coinfection of ferrets with S. pneumoniae and IAV reduced shedding of IAV, instead causing pneumonia and bacteremia (27). Infant mouse models of IAV transmission have shown a reduced shedding of IAV in pups colonized with S. pneumoniae (35) in a sialidase-dependent manner. However, in humans naturally infected with IAV, the diversity and presence of *Neisseria* were shown to increase the duration of IAV shedding (36). Together, these studies support a role for the human upper respiratory microbiome in shedding of pathogenic bacteria and viruses and suggest differential contributions of the microbiome in alterations of pathogen shedding. However, the roles of the microbiota and coinfecting pathogens in the upper respiratory tract on IAV transmission are complicated; they may be host dependent and specific to certain regions of the upper respiratory tract, requiring further study.

Coinfection with multiple respiratory viruses can alter shedding dynamics of each virus. A prior infection with another respiratory virus decreased duration of RSV shedding, but simultaneous infection increased the duration of RSV shedding in a household transmission study (37). In addition, simultaneous coinfection with both RSV-A and RSV-B subtypes or with either RSV-A or RSV-B subtype and another respiratory virus enhances the quantity of RSV shed (38). Coinfection with other respiratory viruses was suggested in SARS superspreading events in 2003 (39) and has been suggested as a mode of enhanced transmission for SARS-CoV2 (40). The mechanism behind respiratory virus coinfection and shedding of each virus is presumably multifactorial, since the virus infection can be immunomodulatory, causing increased mucus production, coughing, or sneezing that could physically expel coinfecting pathogens, or could be coincidental due to common exposure conditions for several respiratory viruses.

Respiratory coinfection in domesticated animals enhances pathogen colonization density (41, 42) and shedding, which has relevance for animal health and welfare, integrity of the food animal supply chain, and control of zoonotic and foodborne infections. Chickens coinfected with low-pathogenicity avian influenza (LPAI) and classical infectious bronchitis virus (a gammacoronavirus) had enhanced shedding of both viruses (43, 44); however, compared to singly infected birds, animals coinfected with variant infectious bronchitis virus had enhanced shedding of only LPAI (43). Mammals with coinfecting respiratory pathogens also had enhanced shedding of IAV, with enhanced shedding of swine influenza seen in piglets coinfected with Actinobacillus pleuropneumoniae and swine influenza (45). In addition, mink exhibit higher shedding of IAV when coinfected with Pseudomonas aeruginosa (46).

Coinfections, likewise, alter pathogen shedding at the genital mucosa. Human immunodeficiency virus infection can increase Neisseria gonorrhoeae and Chlamydia trachomatis bacterial loads in the vagina and cervical mucus of infected people (47). Further, epidemiological connections support the connection between coinfection with HIV and bacterial sexually transmitted infections (STIs) (48, 49). Coinfection with N. gonorrhoeae, likewise, increases vaginal HIV shedding in humanized mice (50), and bacterial STIs, including infections with N. gonorrhoeae, and C. trachomatis, and protozoan pathogen Trichomonas vaginalis enhance shedding of HIV in infected humans (51, 52). However, both bacterial and viral STI acquisition are also dependent on the vaginal microbial community, which complicates the determination of their respective roles in pathogen shedding. Mechanisms for altered shedding are local immunomodulation by vaginal lactobacilli, which reduces genital shedding of HIV without altering systemic viral load (53), and altering vaginal communities in response to concurrent viral infection. HIV-1/HSV-2-coinfected women have higher diversities in the vaginal microbiome, increased anaerobes, decreased lactobacilli, and increased proinflammatory cytokine levels, suggesting enhancement of HIV shedding potential (54). Further, infection with a bacterial STI or presence of bacterial vaginosis enhanced interleukin-10 expression in cervical secretions, allowing increased viral replication and viral load, thereby providing a mechanism for enhanced shedding of HIV (55).

Shedding of GI pathogens is also altered in both humans and domesticated animals by coinfecting pathogens. Shedding of human noroviruses in asymptomatic children is enhanced by coinfection with other enteric viruses (56). Zoonotic hepatitis E virus has enhanced shedding in pigs coinfected with porcine reproductive and respiratory syndrome virus (PRRSV) (57) or porcine circovirus type 2 (58) with a higher hepatitis E viral load in feces and for a longer time than hepatitis E virus monoinfected pigs. Further, both PRRSV and porcine circovirus also enhance transmissibility of hepatitis E between pigs and lead to a higher liver titer of hepatitis E virus, leading to potential zoonotic foodborne infection from eating contaminated liver (59). Coinfection with Lawsonia intracellularis or PRRSV enhances shedding of Salmonella enterica subsp. *enterica* (60), another foodborne illness causative agent related to pork consumption and production. Boiler chickens infected with infectious bursal disease virus have enhanced shedding of Campylobacter jejuni due to immunosuppression by viral infection (61). Cows with liver fluke Fasciola hepatica in their feces have increased likelihood of Shiga toxigenic E. coli in their feces (62). These findings provide further examples of agricultural pathogens enhancing the risk of foodborne illness.

The beneficial gut microbiome has direct and indirect roles in blocking pathogen acquisition, so modifying the members of the community can alter shedding of pathogens. Compared to noncolonized gnotobiotic pigs, gnotobiotic pigs that received a human fecal microbiome transplant have enhanced shedding of human noroviruses (63) and rotaviruses (64), as measured by both more days of shedding and higher titers shed, suggesting a role for the human gut microbial community in viral shedding. Likewise, shedding of live, attenuated rotavirus vaccine in humanized piglets was dependent on microbial community (65), as was shedding of rotavirus vaccine virus in antibiotic-treated adult volunteers (66). Attempts to alter the bacterial community to reduce pathogen shedding have also proven fruitful, as gnotobiotic pigs colonized with probiotic E. coli Nissle strain have reduced rotavirus severity and shedding duration (67). However, supplementing cattle feed with beneficial microbes did not reduce the frequency of shedding nor the amount of E. coli or Salmonella shed (68). Modifying the chicken gut microbiome with live, attenuated S. enterica vaccination or dietary supplementation with galacto-oligosaccharides reduced shedding of virulent S. enterica (69). Disrupting the gut microbiome with antibiotics enhances Clostridioides difficile (70), Klebsiella pneumoniae (71), and Salmonella (72) shedding in mice, supporting a role for the microbiome in preventing hospital-associated and foodborne infections, respectively. Antibiotic treatment led to changes in resident microbiome community composition, supporting the roles of the gastrointestinal microbiome in alteration of shedding and transmission dynamics. Further, modulating the microbiome by supplementing the diet with *Lactobacillus* prevents C. difficile shedding in a mouse model (73).

Pathogen shedding is not consistent across all infected hosts. Some hosts appear to shed pathogen at a much higher frequency, a phenomenon deemed “supershedding”; this, along with other environmental factors, leads to the phenomenon of “superspreading,” where 20% of infections lead to 80% of new infections (74). The microbiome has been implicated in supershedding of Salmonella (70) and enterohemorrhagic E. coli (75) and to immune-mediated supershedding of E. coli (76).

Viruses such as herpesviruses that lead to a chronic infection can be shed throughout the host’s life span. These reactivation and shedding events are often linked to immunosuppressive events and environmental factors. However, coinfecting HIV, beyond being immunosuppressive, has been shown to enhance salivary shedding of human cytomegalovirus, human herpesvirus 8, and Epstein-Barr virus in patients with high HIV titers more than in those with a suppressed HIV load (77). Finally, certain classes of virus require infection by another virus to compete their infection cycles, and thus, to be shed. These viruses include the satellite viruses of plants, adeno-associated viruses of mammals, hepatitis deltavirus, and the recently recognized related deltaviruses of birds (78) and reptiles (79, 80).

## POLYMICROBIAL IMPACTS ON ENVIRONMENTAL SURVIVAL

After being shed, a pathogen must oftentimes survive in the environment until it encounters a new susceptible host to infect. The external environment presents many potential challenges for pathogen survival. In the environment, pathogens interact with other microbes in the air, soil, on biotic and abiotic surfaces, and in and on vector hosts. Interaction with other microbes that are simultaneously shed from the infected host or encountered on biotic and abiotic surfaces can protect the pathogen from desiccation and UV light, as detailed in examples below. Alternatively, inactivation of pathogens can be accelerated by such interactions. For example, peptidoglycan-associated cyclic lipopeptide of Bacillus subtilis destabilizes coronaviruses and other enveloped viruses, suggesting that these viruses may be inactivated or their infectivity may be reduced by association with soil environments (81). Likewise, bacterial lipopolysaccharide (LPS) destabilizes influenza A viruses (82). However, for enteric viruses, interactions with bacteria and their products are stabilizing. Picornaviruses (83–85), reoviruses (86), caliciviruses (87, 88), and astroviruses (89) are stabilized by interactions with bacteria or isolated bacterial envelope components such as peptidoglycan, LPS, or lipoteichoic acid (LTA). This suggests that these enteric viruses may be stabilized during their environmental phase by either the bacterial components of the microbiome of the original host or by environmental bacteria present after shedding.

Recent work has shown similar stabilizing effects from certain members of the human upper respiratory microbial community protecting IAVs from desiccation-mediated viability loss. Again, a component of the bacterial cell envelope—the polysaccharide capsule of both S. pneumoniae and H. influenzae—was shown to be important for stabilizing IAV (34). Further, depletion of the ferret upper respiratory community with antibiotics prior to infection with IAV inhibited respiratory transmission to either antibiotic-depleted or untreated naive contact ferrets. Reconstitution of the donor ferret bacterial community with S. pneumoniae restored respiratory transmission of IAV to naive untreated contact animals (34). This finding supports a role for the host’s bacterial community in infection by viral pathogens. Another respiratory pathogen, the opportunistic bacterial pathogen Legionella pneumophila, which is acquired through inhalation of contaminated water, has enhanced environmental survival when internalized by fungi, protecting the bacteria from UV light (90).

The microbiome of vector hosts also has a key role in transmission of bacterial, parasitic, and viral pathogens. Best characterized is the role of the mosquito endosymbiont Wolbachia pipientis in preventing infection of mosquitoes and therefore the transmission of arboviruses (91) and *Plasmodium* (91). Proposed mechanisms of viral inhibition include modulation of lipid metabolism in the mosquito host (92), direct blockade of viral entry into mosquito cells (93), and degradation of viral RNA (94). Another mosquito commensal, *Chromobacterium* sp. Panama, present in Aedes aegypti midgut and the soil, secretes a protease that degrades dengue virus envelope protein, thereby blocking infection of mosquito cells *in vitro* and *in vivo* (95) and transmission of dengue virus to human hosts. The natural microbiome of the Ixodes scapularis tick is necessary for the tick’s colonization by Borrelia burgdorferi, the causative agent of Lyme disease (96). Endosymbionts of Dermacentor andersoni ticks can control infection and transmission of Anaplasma marginale, a rickettsial pathogen of livestock, and Francisella novicida, a bacterial pathogen related to the causative agent of tularemia (97).

Foodborne illness is a leading cause of morbidity and mortality worldwide. In addition to the above-mentioned effects of coinfection and microbiome composition of animal hosts in shedding of pathogens in animals used for food, large outbreaks are increasingly being traced back to animal or human fecal contamination of produce, either directly or through the irrigation water (98). Human and animal feces, where one-quarter to one-half of the dry mass is bacterial in origin (99) stabilize enteric viruses on environmental fomites (100). The endogenous microbiome of the plant and associated soil also stabilize pathogens or promote internalization of the pathogen into the plant cell (101), where it can be protected from decontamination. The most common foodborne viruses are hepatitis A virus and norovirus. Both are nonenveloped and have high environmental stability. Peptidoglycan from Bacillus subtilis, found in soil and plant phyllosphere environments, protected a norovirus surrogate from bleach-mediated decontamination (102) even in the absence of direct binding of virus to the bacterial surface. Murine norovirus (103) and human noroviruses (88) directly interact with bacteria (88, 103) and fungi (103) and therefore might be protected from decontamination to a higher degree. Bacterial burden and damage to plant tissues correlated with higher norovirus stability on harvested leaves (104), suggesting that bacterial members of the leaf microbiome contribute to norovirus persistence and that this can be enhanced by harvesting or bacterial-mediated damage to the leaf tissues.

Shellfish are also a common source of foodborne infection. Since they are often eaten raw or undercooked, the role of the normal flora of shellfish on pathogen colonization is critical to shellfish-borne infection because high heat inactivation cannot protect consumers in these cases. Shellfish can concentrate foodborne pathogenic viruses from human and animal fecal contamination of seawater within their tissues, including norovirus (105), hepatitis A virus (106), and hepatitis E virus (107). Human pathogens such as Vibrio vulnificus and V. parahaemolyticus are normal commensals of oysters (108, 109). Further, interactions between normal commensals of oysters with S. enterica *in vitro* can activate quorum-sensing virulence regulons (110), providing a further mechanism for foodborne-associated illness from shellfish.

## POLYMICROBIAL IMPACTS ON ACQUISITION

Probably the best-characterized role of the microbiome in transmission is that of prevention of infection in the new host. Much effort has gone into understanding these protective microbial communities and modifying them via the ingestion and application of probiotics (111). Characterization of the microbial community in people with or without respiratory infection (36, 112–115) and characterization of beneficial and detrimental vaginal microbial communities for bacterial and viral pathogens (116–121) have been insightful for predicting infection risk. Predicted beneficial interactions are both indirect, through bacterial modulation of immune function and epithelial barrier integrity, and direct, through prevention of pathogen binding to epithelial tissues, depletion of metabolites, and production of antagonistic metabolites. The main method by which polymicrobial interactions can alter acquisition of other pathogens is by changing the local inflammatory state, thereby increasing or decreasing the likelihood of successful infection. In addition, the resident microbial community plays an important role in maintaining the permeability of epithelial barriers, alteration of which can impact the invasive potential of pathogens. Nutrient availability, toxic metabolites, and signaling molecules produced by the microbial community at the site of infection alter the pathogen’s ability to grow and to express virulence-associated molecules or the host tissue’s ability to alter receptor expression. Together, these factors produced by the infection site’s microbial community alter the pathogen’s ability to successfully infect its next host.

Immune modulation can act either locally or systemically and can be protective or can enhance infection susceptibility. Induction of interferon (IFN) lambda by gut commensal bacteria can lead to persistent murine norovirus infection (122). IFN lambda can also act in the nasal mucosa, where its induction by IAV can promote Staphylococcus aureus and Streptococcus pneumoniae colonization and infection (123); however, Staphylococcus epidermidis can act on nasal cells to produce IFN lambda and prevent IAV infection (124). Local production of IFN beta in the gut stimulated by *Bacteroidetes* outer membrane vesicle glycolipids can block experimental infection with vesicular stomatitis virus and may act more broadly to provide antiviral resistance in the gut (125). Immunomodulatory signals by the gut microbial community can also alter susceptibility to nonenteric infections. Production of the short-chain fatty acid butyrate by enteric commensals can lead to a systemic suppression of IFN production and interferon responsive gene expression and increase susceptibility to influenza virus, reovirus, HIV-1, human metapneumovirus, and vesicular stomatitis virus (126). Alteration of the gut microbial community during an IAV infection can alter the production of another short-chain fatty acid, acetate, reducing bactericidal activity of alveolar macrophages and enhances susceptibility to bacterial pneumonia (127).

Another beneficial function of the microbiota in preventing infection is the promotion of epithelial barrier integrity (128), with microbial signals promoting healthy tissue function by increasing expression of cell-cell adhesion molecules and modulating inflammatory signals. While typically characterized in the mucosa of the gut (129), the microbiota of the skin is important in maintaining barrier integrity and preventing Staphylococcus aureus infection (130). In the vagina, Lactobacillus crispatus colonization can promote epithelial cell growth and barrier integrity (131), but Gardnerella vaginalis, Atopobium vaginae, and Prevotella bivia, which are considered “bad” vaginal microbiota, act to disrupt tight junctions and can promote the acquisition of the protozoan pathogen Trichomonas vaginalis (132).

Barrier function in the respiratory tract is enhanced by mucociliary clearance. The human disease cystic fibrosis results in altered viscosity of mucus and reduced mucociliary clearance, enhancing susceptibility to bacterial colonization and infection of the lower respiratory tract. Alterations of mucociliary clearance can also be bacterially mediated. Colonization of porcine lung tissues with Bordetella bronchiseptica led to reduced ciliary action and enhanced susceptibility to colonization and infection with Streptococcus suis (133).

Production of toxic metabolites is another mechanism by which the commensal microbiota prevent colonization and infection by pathogens. A secreted product of the human commensal Lactobacillus reuteri, known as reuterin, leads to production of reactive oxygen species by Clostridioides difficile to reduce C. difficile outgrowth (134). Microbiota-derived fatty acids, in addition to their roles in immunomodulation described above, can alter pathogen colonization and infection. The intestinal parasite Cryptosporidium parvum is inhibited by medium- or long-chain saturated fatty acids derived from microbiota, and yet long-chain unsaturated fatty acids enhanced the invasive potential (135) of the parasite. In addition to microbial modulation of C. difficile growth, *Bloutia* and *Clostridium sporogenes* metabolites can reduce toxin expression by C. difficile (136, 137). In addition, glycan and sphingolipid metabolism by the gut microbiota can alter susceptibility to human norovirus infection (138).

The microbiota or coinfecting pathogens can alter host cell receptor expression to both enhance and inhibit pathogen infection. Colonizing Streptococcus pneumoniae can inhibit IAV infection when bacterial sialidase removes terminal sialic acid residues used for viral attachment and infection (35). Neisseria gonorrhoeae infection stimulates cytokine production, increasing both immune cell trafficking to the cervix and expression of viral receptors enhancing HIV infection (139).

Quorum-sensing signals from coinfecting pathogens or cytokine signals from infected hosts—even those infected with a different pathogen—can alter bacterial behavior and lead to enhanced pathogenesis. Nontypeable Haemophilus influenzae upregulates pili in virally coinfected cells, increasing colonization and invasive potential (140). Pathogen behavior can also be changed through promotion or inhibition of biofilm formation and by formation of multispecies biofilms in the middle ear (141) or gut (142), which can promote both pathogenesis and antimicrobial treatment failure. Multispecies biofilms can even be trans-kingdom, with Staphylococcus and *Candida* (143) and *Enterococcus* and *Candida* (144) forming multispecies biofilms. In addition to biofilm-mediated antimicrobial therapy failure, antimicrobial resistance can be transferred from one pathogen to another, particularly in the multispecies biofilm, but also in other cocolonization scenarios, such as Streptococcus agalactiae obtaining antimicrobial resistance from coinfecting sexually transmitted pathogens (145).

## CONCLUSIONS

Polymicrobial interactions can enhance or reduce pathogen transmission. Greater understanding of the roles of normal and pathogenic members of the microbiome, mycobiome, and virome on transmission of pathogens can lead to insights into control of both endemic and epidemic transmission. Linking certain natural or altered microbial communities to “supershedding” events could explain spillover events in zoonotic transmission and the asymmetric shedding seen in epidemics when 20% of cases cause 80% of new cases (74). In addition, identifying how the pathogens evolved to exploit each other and the microbiome can lead to greater understanding of host-pathogen interactions. As we better characterize the various human, plant, and animal microbial communities, we can decipher the common mechanisms by which pathogens exploit these communities to both establish infection and to be shed and survive in the environment to establish a new infection. Further, knowing the interactions between pathogens and the microbiome of native and nonnative hosts could lend insights into zoonotic transmission.
